# Heart sound classification based on convolutional neural network with convolutional block attention module

**DOI:** 10.3389/fphys.2025.1596150

**Published:** 2025-06-05

**Authors:** Ximing Huai, Lei Jiang, Chao Wang, Peng Chen, Hanchi Li

**Affiliations:** ^1^ Ningbo Key Laboratory of Intelligent Manufacturing of Textiles and Garments, Zhejiang Fashion Institute of Technology, Ningbo, Zhejiang, China; ^2^ Laboratory of Intelligent Home Appliances, College of Science and Technology, Ningbo University, Ningbo, Zhejiang, China; ^3^ Fudan Institute on Ageing, Fudan University, Shanghai, China; ^4^ School of Design Media, Zhejiang Fashion Institute of Technology, Ningbo, Zhejiang, China; ^5^ School of Textile, Zhejiang Fashion Institute of Technology, Ningbo, Zhejiang, China

**Keywords:** heart sound classification, convolutional neural network, convolutional block attention module, CBAM, attention mechanism, medical signal processing

## Abstract

Cardiovascular diseases (CVDs) remain a leading cause of global mortality, underscoring the need for accurate and efficient diagnostic tools. This study presents an enhanced heart sound classification framework based on a Convolutional Neural Network (CNN) integrated with the Convolutional Block Attention Module (CBAM). Heart sound recordings from the PhysioNet CinC 2016 dataset were segmented and transformed into spectrograms, and twelve CNN models with varying CBAM configurations were systematically evaluated. Experimental results demonstrate that selectively integrating CBAM into early and mid-level convolutional blocks significantly improves classification performance. The optimal model, with CBAM applied after Conv Blocks 1-1, 1-2, and 2-1, achieved an accuracy of 98.66%, outperforming existing state-of-the-art methods. Additional validation using an independent test set from the PhysioNet 2022 database confirmed the model’s generalization capability, achieving an accuracy of 95.6% and an AUC of 96.29%. Furthermore, T-SNE visualizations revealed clear class separation, highlighting the model’s ability to extract highly discriminative features. These findings confirm the efficacy of attention-based architectures in medical signal classification and support their potential for real-world clinical applications.

## 1 Introduction

Cardiovascular disease (CVD) remains a leading global health challenge, accounting for one-third of all deaths worldwide, with 85% of these deaths attributable to heart attacks and stroke (Cardiovascular diseases CVDs, 2025). The global burden of CVD is exacerbated by two significant trends: the aging population and the increasing prevalence of CVD among younger demographics (Herrington et al., 2016). Particularly in low- and middle-income countries, CVD not only contributes to high mortality rates but also imposes substantial economic burdens on healthcare systems and families (Wurie and Cappuccio, 2012). These pressing concerns underscore the critical need for effective early detection and diagnostic methods.

**FIGURE 1:**
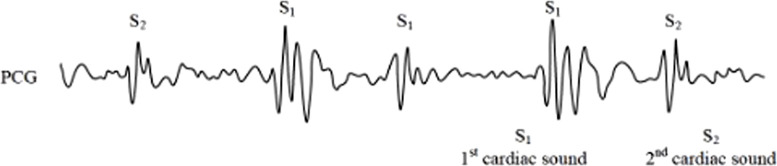
Phonocardiogram (PCG) signals.

Cardiac auscultation, as a non-invasive diagnostic technique with a history spanning over 180 years, continues to play a crucial role in cardiovascular assessment (Abbas and Bassam, 2009). The phonocardiogram (PCG), which captures the mechanical activity of the heart including atrial and ventricular function as well as major vessel status, provides valuable diagnostic information. The fundamental heart sounds, S1 and S2, correspond to isovolumetric ventricular systole and diastole respectively (Figure 1). However, the clinical interpretation of PCG signals presents several challenges: (1) it requires substantial clinical expertise, (2) human auditory perception has limited sensitivity across different frequency ranges, and (3) subjective interpretation may lead to diagnostic variability. These limitations have driven significant research interest in developing automated computer-aided analysis and classification systems for heart sound signals.

Although state-of-the-art research has achieved significant progress, there is still a need to improve detection accuracy in order to enable earlier diagnosis and increase survival rates. CBAM has demonstrated remarkable success in image classification and detection tasks by enabling networks to focus on relevant spatiotemporal features. Building on this strength, the present study aims to maximize classification accuracy by combining convolutional neural networks with attention mechanisms to enable automated feature extraction. This paper makes three primary contributions to the field of automated heart sound classification:

(1) We propose and validate a novel CNN + CBAM framework for heart sound classification, demonstrating significant improvements in classification accuracy compared to existing approaches using the same dataset.
(2) We systematically investigate the optimal integration strategy for CBAM within CNN architectures, revealing that selective rather than comprehensive CBAM implementation yields superior performance.
(3) We demonstrate the potential of attention mechanisms in medical signal analysis, particularly in scenarios requiring automated feature extraction and emphasis on critical signal components.

The remainder of this paper is organized as follows: Section 2 summarizes the relevant works. Section 3 describes the datasets, preprocessing procedures, proposed model architecture, and evaluation methodology. Section 4 presents the experimental results, including ablation studies and comparative analyses with existing approaches. Section 5 discusses the key findings and their implications. Finally, Section 6 concludes the paper and outlines directions for future research.

## 2 Related work

Automated heart sound classification methods can be broadly categorized into traditional machine learning approaches and more recent deep learning-based techniques. A brief review of recent advances in automated heart sound classification is provided in this Section.

### 2.1 Traditional and early deep learning approaches

Earlier approaches to heart sound classification relied heavily on manual feature extraction and conventional classifiers. Nogueira et al. (2019) combined temporal and Mel-frequency cepstral coefficient (MFCC) features with Support Vector Machine (SVM) classification, while Hamidi et al. (2018) proposed innovative feature extraction methods combining curve fitting with MFCC and fractal features. Homsi et al. (2016) applied SMOTE for class imbalance handling and tested multiple ensemble classifiers on handcrafted features. While these methods have demonstrated potential, they are inherently limited by their dependence on labor-intensive segmentation and expert-driven feature selection, where any inaccuracies can substantially compromise classification performance.

The emergence of CNNs has significantly improved performance by enabling end-to-end learning. Meintjes et al. (2018) utilized continuous wavelet transforms and CNNs for heart sound classification, while Potes et al. (2016) fused hand-crafted features and CNN outputs in an ensemble framework. Chen et al. (2020) and Li et al. (2020) enhanced CNN architectures with improved time-frequency representations and pooling mechanisms, respectively. These efforts marked a transition from manual feature engineering to deep feature learning.

Despite these advancements, CNN-based approaches face several challenges in heart sound classification: (1) high data requirements, particularly problematic given the typically small and imbalanced nature of medical datasets; (2) insufficient attention mechanisms, leading to potential learning of irrelevant features; and (3) limited generalizability and interpretability, which are crucial for clinical applications.

### 2.2 Recent advances in CNN-Based methods

From 2020 to 2025, heart sound classification has undergone significant methodological evolution, transitioning from basic CNN-based pipelines to hybrid deep learning frameworks that integrate attention mechanisms, temporal modeling, and signal decomposition strategies.

CNN architectures initially dominated the field for their ability to extract local time-frequency features from phonocardiograms (PCG). Cheng et al. (2019) proposed a lightweight CNN optimized for mobile use, achieving high performance on the PhysioNet CinC 2016 dataset. Chen et al. (2019) used ensemble CNNs to classify unsegmented PCG, demonstrating robustness under clinical constraints.

Recent work has moved toward hybrid models that enhance CNNs with attention mechanisms, such as Squeeze-and-Excitation (SE), CBAM, and Transformer-based modules. While CBAM focuses on spatial and channel refinement, SE blocks (Hu et al., 2018) provide channel recalibration with minimal overhead, and Transformer structures (Vaswani et al., 2017) offer superior global context modeling. Marques & Oliveira (Marques and Oliveira, 2025) showed that Transformer-based ECG classifiers could outperform CNNs in capturing long-range dependencies, and Rahman (Rahman, 2025) proposed a CNN-Transformer fusion for ECG, demonstrating state-of-the-art accuracy. Recently, hybrid frameworks combining convolutional architectures with Transformer encoders have emerged as promising solutions for heart sound classification. For example, Al-Tam et al. (2024) proposed a hybrid model integrating a Transformer encoder with residual convolutional blocks for cardiovascular disease recognition using heart sounds. This approach leverages the Transformer’s capability to model long-range dependencies alongside the local feature extraction strengths of convolutional networks, achieving superior classification performance. Such architectures highlight the trend toward incorporating both global and local feature modeling in medical signal analysis.

Another growing direction involves feature extraction techniques beyond spectrograms. Inspired by ECG analysis, methods such as wavelet packet decomposition (WPD), empirical mode decomposition (EMD), and variational mode decomposition (VMD) have been applied to biomedical signals to isolate diagnostically relevant components. These techniques are increasingly used as front-ends to deep models, enriching feature space and improving performance in noisy or low-resource settings (Vocaturo and Zumpano, 2021).

A critical issue raised in recent reviews is dataset diversity. Models trained solely on the CinC 2016 dataset may suffer from poor generalizability. Khalid et al. (2024) emphasized that inter-patient variability, device differences, and class imbalance can significantly affect performance. Approaches such as domain adaptation, federated learning, and data augmentation (including GAN-based synthetic signal generation) have emerged to address these issues and enhance robustness across real-world scenarios.

## 3 Materials and methods

This section briefly introduces the dataset and preprocessing steps, describes the architecture of the Convolutional Neural Network (CNN), and explains how the Convolutional Block Attention Module (CBAM) is integrated. The final part outlines the experimental design, including model configurations and evaluation procedures.

### 3.1 Dataset and data preprocessing

The dataset used in this study is derived from the 2016 PhysioNet Computing in Cardiology Challenge on Classification of Heart Sound Recordings (Liu et al., 2016). It comprises six sub-datasets (training-a to training-f), containing a total of 3,240 heart sound samples, including 2,575 normal and 665 abnormal recordings. Each sample is a heart sound signal with a sampling rate of 2 kHz and a duration ranging from 5 to 122 s. All samples are labeled by category and stored in. wav format. The detailed distribution of samples across sub-datasets is provided in Table 1.

**TABLE 1:** Database statistics.

Dataset	Sample size		
	Normal	Abnormal	Total
training-a	117	292	409
training-b	386	104	490
training-c	7	24	31
training-d	27	28	55
training-e	1,958	183	2,141
training-f	80	34	114
Total	2,575	665	3,240

**FIGURE 2:**
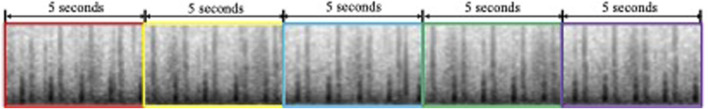
Heart Sound segmentation.

To standardize input length and ensure comprehensive diagnostic coverage, each heart sound recording was divided into fixed, non-overlapping 5-second intervals (Figure 2). This duration, supported by clinical consultation and prior studies (Cheng et al., 2019), reliably captures at least one full cardiac cycle (typically 2–3 s), which is sufficient to include pathological features such as murmurs and arrhythmias. All available full-length segments were extracted from each recording to maximize information retention, rather than selecting a single portion. Recordings shorter than 5 s and incomplete trailing segments were excluded to avoid boundary artifacts and preserve signal integrity. Clinical experts confirmed that pathological patterns generally recur across the signal, making them likely to appear in at least one of the segments. This segmentation strategy achieves a practical balance between input uniformity, diagnostic completeness, and computational efficiency, making it well-suited for CNN-based classification tasks.

**FIGURE 3:**
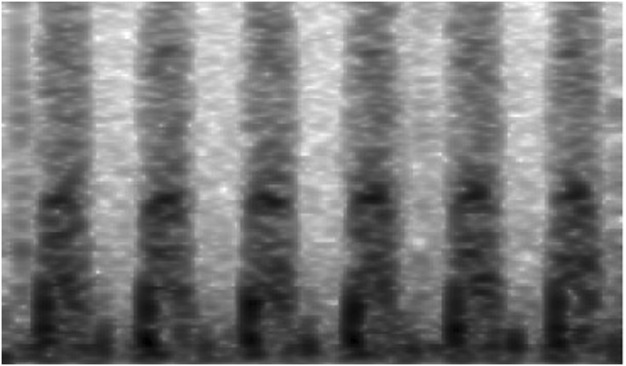
Spectrogram of abnormal heart sounds.

**FIGURE 4:**
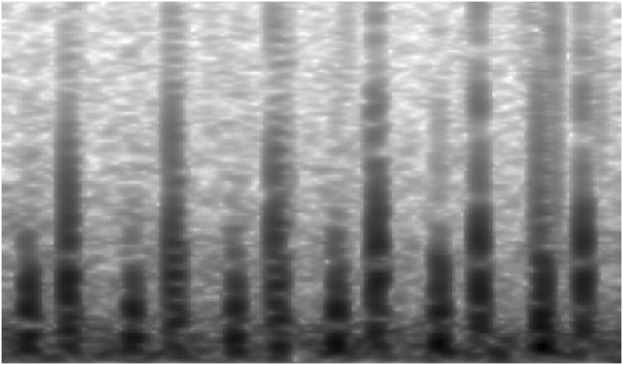
Spectrogram of normal heart sounds.

Each valid segment was then converted into a grayscale spectrogram of size 864 × 504 pixels using Python’s audio processing libraries (e.g., wave, NumPy). Grayscale representation was adopted to simplify color channels while preserving critical amplitude and frequency features. Examples of spectrograms for normal and abnormal heart sounds are shown in Figures 3, 4, respectively. This preprocessing resulted in a total of 12,378 spectrograms. The spectrograms were used for 10-fold cross-validation, with the data partitioned at a 9:1 ratio into training and validation sets for model development and evaluation.

### 3.2 Convolutional neural network (CNN)

Convolutional Neural Networks (CNNs) are a class of deep learning models particularly well-suited for image-like data due to their ability to extract hierarchical spatial features through convolutional operations (Browne and Ghidary, 2003). A typical CNN architecture consists of multiple convolutional layers followed by pooling layers and fully connected layers, enabling automatic feature learning and classification (Mo et al., 2019).

In the context of heart sound classification, CNNs have demonstrated remarkable performance. For instance, Wu et al. (2019) proposed a CNN-based method for phonocardiogram classification, achieving a sensitivity of 86.46% and specificity of 85.63% in holdout testing. Rubin et al. (2016) combined heart sound segmentation with time-frequency heat maps and a CNN, attaining an accuracy of 83.4%. Chen et al. (2019) employed a CNN to classify unsegmented PCG signals, reporting a sensitivity of 92.73%, specificity of 96.90%, and mean accuracy (MACC) of 94.81%. Cheng et al. (2019) further optimized CNN architectures for mobile-device deployment, achieving a recognition rate of 96.16%. Lee and Kwak (2023) proposed a method for feature extraction using Wavelet Scattering Transform (WST) and Continuous Wavelet Transform (CWT), followed by heart sound classification using 1D and 2D Convolutional Neural Networks (CNNs). Experimental results showed that the 1D-CNN and 2D-CNN models achieved accuracy rates of 96.1% and 95.29%, respectively, on the PhysioNet/CinC 2016 dataset. These studies collectively highlight the robustness of CNNs for heart sound classification tasks.

### 3.3 Convolutional block attention module (CBAM)

**FIGURE 5:**
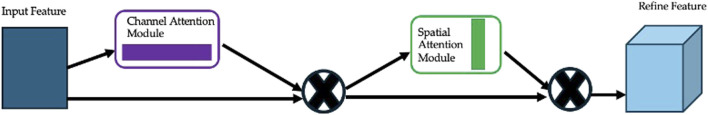
The overview of CBAM.

The Convolutional Block Attention Module (CBAM) (Woo et al., 2018) is a lightweight, yet effective module designed to enhance feature representations in CNNs by applying attention mechanisms along both channel and spatial dimensions. As illustrated in Figure 5, CBAM consists of two sequential submodules: channel attention and spatial attention.

**FIGURE 6:**
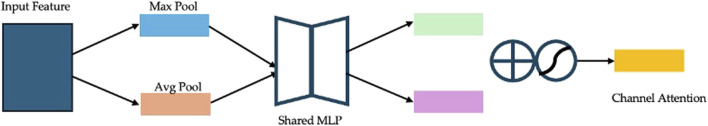
Diagram of channel attention module.

**FIGURE 7:**
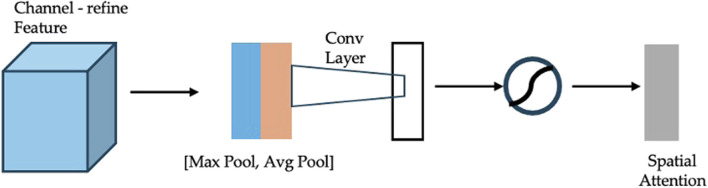
Diagram of spatial attention module.

(1) Channel Attention: The channel attention submodule (Figure 6) generates an attention map by aggregating spatial information through global average pooling and global max pooling. The pooled features are processed by a shared multi-layer perceptron (MLP), and their element-wise summation is activated by a sigmoid function to produce the channel attention map. This map is then multiplied elementwise with the input feature map to emphasize important channels.
(2) Spatial Attention: The spatial attention submodule (Figure 7) refines the feature map further by compressing channel information through global average and max pooling. The pooled features are concatenated and processed by a convolutional layer followed by a sigmoid activation to generate the spatial attention map. This map is multiplied elementwise with the channel-refined feature map to highlight relevant spatial regions.

CBAM’s lightweight design allows it to be seamlessly integrated into various CNN architectures with minimal computational overhead. Previous studies have demonstrated its effectiveness in diverse applications. For example, Boyu et al. (Chen B. et al., 2020) incorporated CBAM into a 3D CNN for micro-expression recognition, outperforming state-of-the-art methods. Zhang et al. (2021) enhanced COVID-19 diagnosis accuracy using a CBAM-based attention network, while Zhang and Wang (2022) achieved efficient finger vein recognition with a lightweight CNN + CBAM architecture.

### 3.4 Experimental design and evaluation

#### 3.4.1 Network architecture

**FIGURE 8:**
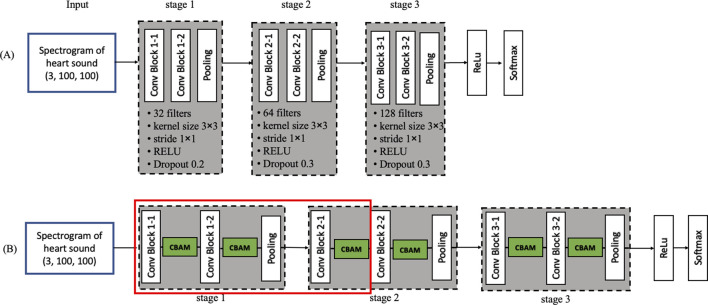
Convolutional neural network diagram **(A)** Model 1: Base convolutional neural network **(B)** Model 12.

The base CNN used in this study (Figure 8A) is adapted from previous work (Huai et al., 2021) and organized into five stages: three convolutional stages, one fully connected stage, and a final SoftMax classification stage. Each convolutional stage contains two convolutional layers, yielding six in total (Conv Block 1-1, 1-2, 2-1, 2-2, 3-1, and 3-2). Specifically, first stage, 32 filters per convolutional layer (kernel size 3 × 3, stride 1 × 1), followed by 2 × 2 max pooling and RELU activation with a dropout rate of 0.2. Second stage, 64 filters, otherwise similar to the first. Third stage, 128 filters, again following the same pooling, activation, and dropout settings. A fully connected layer (output dimension = 500) with RELU activation and a dropout rate of 0.3 is added before the SoftMax layer, which classifies heart sounds into normal or abnormal. The learning rate is controlled using the ReduceLROnPlateau function (monitor = 'val_loss’, factor = 0.5, patience = 5, verbose = 1, min_lr = 1e-7). The optimizer used is Adam with a learning rate of 0.001. The batch size for training is set to batch_size = 10. To prevent overfitting during training, an early stopping strategy was adopted. The training process was monitored using validation loss, and training was terminated if no improvement was observed for 15 consecutive epochs. This ensured that the model maintained optimal generalization capability without excessive training.

Regarding the choice of activation function, we employed the SoftMax function for classification, despite sigmoid being a common choice in binary classification tasks. SoftMax was selected primarily for its compatibility with the categorical cross-entropy loss function, which enhances numerical stability during training. Additionally, SoftMax offers greater scalability for multiclass problems, should the classification task be extended in the future. We also evaluated the sigmoid function and found that its performance was very similar to that of SoftMax. Specifically, when keeping all other parameters unchanged, the two activation functions yielded nearly identical accuracy, F1 score, and AUC. However, the convergence curve of SoftMax was smoother, indicating more stable training convergence.

The parameters of the network architecture reflect the optimal results after training and adjustment with the dataset used in this study. The ReLU function was used as the activation function due to its advantages in increasing network non-linearity, preventing gradient vanishing, and promoting sparsity in the network.

#### 3.4.2 CBAM integration

To investigate the impact of CBAM, we inserted CBAM modules after different convolutional layers of the base CNN, generating 12 distinct models (Table 2). Model 1 serves as the baseline (i.e., no CBAM), while Models 2–12 progressively incorporate CBAM in various layers. For example, Model 2 adds CBAM only after Conv Block 1-1, whereas Model 12 integrates CBAM into all convolutional layers (Figure 8B). This systematic approach allows a comprehensive assessment of how attention mechanisms influence heart sound classification performance.

**TABLE 2:** Specific parameters of CBAM in 12 models.

Model	Stage 1		Stage 2		Stage 3	
	Conv block 1-1 with CBAM	Conv block 1-2 with CBAM	Conv block 2-1with CBAM	Conv block 2-2 with CBAM	Conv block 3-1with CBAM	Conv block 3-2 with CBAM
1	✕	✕	✕	✕	✕	✕
2	✓	✕	✕	✕	✕	✕
3	✕	✓	✕	✕	✕	✕
4	✓	✓	✕	✕	✕	✕
5	✕	✕	✓	✕	✕	✕
6	✕	✕	✕	✓	✕	✕
7	✓	✓	✓	✕	✕	✕
8	✓	✓	✓	✓	✕	✕
9	✕	✕	✕	✕	✓	✕
10	✕	✕	✕	✕	✕	✓
11	✓	✓	✓	✓	✓	✕
12	✓	✓	✓	✓	✓	✓

#### 3.4.3 Evaluation metrics and setup

All models are trained and evaluated under identical conditions to ensure a fair comparison. The accuracy (ACC), cross-entropy loss, sensitivity (symbol: se), precision, F1_score, and other evaluation metrics are recorded. Among them, abnormal heart sound signals that are correctly classified are true positive (TP) (symbol: TP); abnormal heart sound signals that are incorrectly classified are false positive (FP) (symbol: FP); normal heart sound signals that are correctly classified are true negative (TN) (symbol: TN); and normal heart sound signals that are incorrectly classified are false negative (FN) (symbol: FN). The calculations for se, acc, precision, and F1_score are shown in Formulas 1–4. Training is conducted using deep learning framework TensorFlow, with consistent batch size, learning rate, and number of epochs across all models.

$$Accuracy=\frac{TP+TN}{TP+TN+FP+FN}$$ (1)

$$Precision=\frac{TP}{TP+FP}$$ (2)

$$Recall=\frac{TP}{TP+FN}$$ (3)

$$F1_{score}=\frac{2\left(Precision\times Recall\right)}{Precision+Recall}$$ (4)

Additionally, t-Distributed Stochastic Neighbor Embedding (T-SNE) is applied to project high-dimensional features into a lower-dimensional space for visualization. In binary classification, T-SNE assists in evaluating the model’s ability to distinguish between classes. This study uses T-SNE to examine the clustering and separability of normal and abnormal heart sounds, providing insights into feature learning and highlighting areas prone to misclassification.

## 4 Results

This section presents and discusses the outcomes of the twelve experimental models (Models 1–12) for heart sound classification, followed by a comparison with other related studies. The results underscore the effect of integrating the Convolutional Block Attention Module (CBAM) into different parts of a Convolutional Neural Network (CNN).

### 4.1 Comparison among the twelve models

**FIGURE 9:**
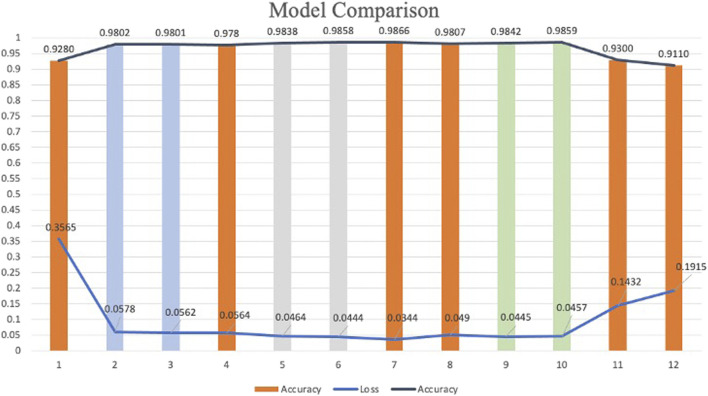
Classification models comparison.

According to the experimental design, twelve models were developed for heart sound classification, each with varying configurations of CBAM integration. The accuracy, loss, Recall, Precision, F1_score, AUC and T-SNE values for each model are summarized in Table 3, Intra_class_0 represents the intra-class distance of normal heart sounds, intra_class_1 represents the intra-class distance of abnormal heart sounds, and inter_class_center_distance refers to the inter-class distance between the normal and abnormal heart sound classes. Also, a comparative analysis of their classification performance is illustrated in Figure 9.

**TABLE 3:** Evaluation indicators of 12 models.

Model	Accuracy	Loss	Sensitivity (recall)	Precision	F1_score	AUC	intra_class_0	intra_class_1	inter_class_center_distance
1	0.9280	0.3565	0.8616	0.8646	0.8631	0.9535	34.4758	18.2857	51.6779
2	0.9802	0.0578	0.9685	0.9412	0.9594	0.9771	35.6107	14.2065	56.1283
3	0.9801	0.0562	0.9817	0.9472	0.9642	0.9835	35.2484	14.4696	61.6872
4	0.978	0.0564	0.9571	0.9470	0.9520	0.9781	34.0582	14.4032	70.3928
5	0.9838	0.0464	0.9826	0.9496	0.9658	0.9817	31.1970	15.4810	66.0397
6	0.9858	0.0444	0.9889	0.9572	0.9728	0.9849	35.4437	15.1191	38.4579
7	0.9866	0.0344	0.9748	0.9679	0.9713	0.9973	36.1278	14.4496	72.0167
8	0.9807	0.0490	0.9661	0.8962	0.9298	0.9697	36.4983	18.4495	41.2070
9	0.9842	0.0445	0.9833	0.9704	0.9768	0.9822	30.7837	14.5418	57.9523
10	0.9859	0.0457	0.9846	0.9772	0.9810	0.9856	33.1949	16.5026	45.3366
11	0.9300	0.1432	0.9182	0.9611	0.9392	0.9236	35.9540	20.9502	35.6403
12	0.9110	0.1915	0.7298	0.8927	0.8030	0.9272	36.3183	25.3992	41.1211

The results demonstrate that models incorporating CBAM (Models 2–11) generally outperform the baseline model (Model 1), which lacks CBAM. This improvement can be attributed to the dual attention mechanisms of CBAM: (1) Spatial Attention, this mechanism enables the network to focus on pixel regions in the spectrogram that are most relevant to classification while ignoring less informative areas; (2) Channel Attention, this mechanism optimizes the allocation of feature map channels, ensuring that the network prioritizes the most discriminative features. The simultaneous application of spatial and channel attention enhances the model’s ability to extract and utilize critical features, leading to higher classification accuracy.

**FIGURE 10:**
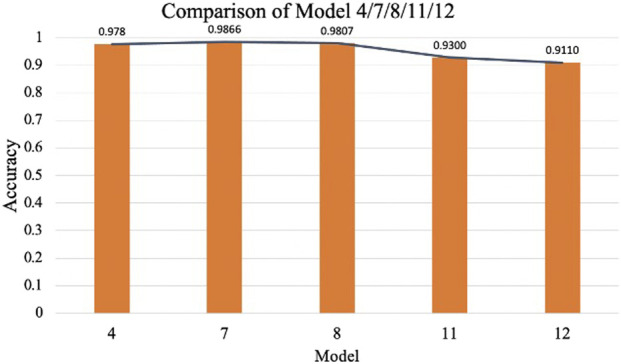
Comparison of model 4/7/8/11/12.

To investigate the effect of CBAM placement, Models 2–12 were designed with CBAM integrated into different convolutional layers. Single-CBAM Models (Models 2, 3, 5, 6, 9, and 10), these models incorporate CBAM in only one convolutional layer within a specific stage. The accuracy differences among these models are minimal, with Model 10 achieving the highest accuracy (0.9859). This suggests that adding CBAM to deeper convolutional layers may yield slightly better performance by emphasizing more abstract feature representations. Multi-CBAM Models (Models 4, 7, 8, 11, and 12), these models incorporate multiple CBAM modules, ranging from two to six. As shown in Figure 10, the accuracy initially increases with the number of CBAM modules but declines after reaching a peak. Model 7, which integrates CBAM in Conv Block 1-1, 1-2, and 2-1, achieves the highest accuracy (0.9866). In contrast, Model 12, which includes CBAM in all convolutional layers, exhibits the lowest accuracy (0.9110). This indicates that while CBAM enhances feature extraction, excessive use can lead to overfitting or reduced generalization capability.

The results suggest that the strategic placement of CBAM in early to mid-level convolutional layers (e.g., Conv Block 1-1, 1-2, and 2-1) is optimal for improving classification performance. This configuration allows the model to amplify relevant features without introducing unnecessary complexity or overfitting.

### 4.2 Training and validation curves of selected models

**FIGURE 11:**
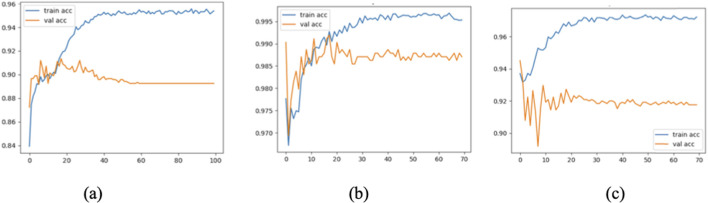
Accuracy curves of Model 1 **(a)**, Model 7 **(b)**, and Model 12 **(c)**.

**FIGURE 12:**
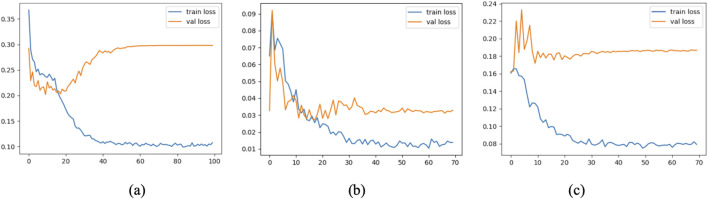
Loss curves of Model 1 **(a)**, Model 7 **(b)**, and Model 12 **(c)**.

To further assess the convergence and generalization capabilities during model training, three representative models were selected: Model 1 (baseline without CBAM), Model 7 (optimal model), and Model 12 (model with CBAM integrated into all convolutional layers). Their training and validation Accuracy and Loss curves are plotted in Figures 11, 12.

As shown in the figure, Model 7 exhibits steadily increasing Accuracy and decreasing Loss on both the training and validation sets, with consistent trends between them, indicating good convergence and generalization. In contrast, Model 1 converges more slowly, and its validation Accuracy remains consistently lower than that of Model 7. Model 12, although achieving high training Accuracy rapidly, shows a significant decline in validation Accuracy and an increase in validation Loss, suggesting overfitting due to excessive CBAM integration.

These results confirm that moderately and strategically incorporating CBAM modules (as in Model 7) can significantly enhance classification performance while mitigating the risk of overfitting.

### 4.3 T-SNE visualization analysis of different models

To gain deeper insight into the feature distribution learned by each model, t-distributed stochastic neighbor embedding (T-SNE) was employed to project the high-dimensional features extracted from the last convolutional layer into a two-dimensional space. This visualization allows for an intuitive assessment of the separability between normal and abnormal heart sound classes.

**FIGURE 13:**
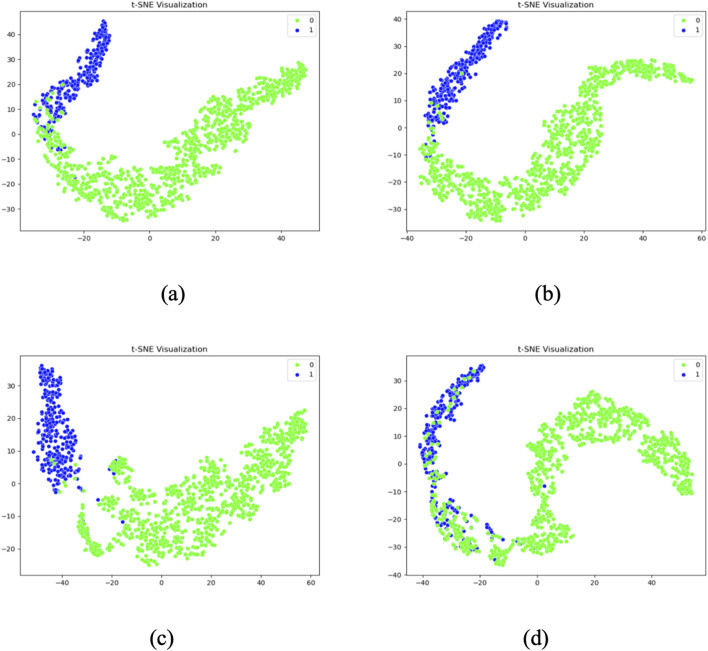
T-SNE visualization results of Model 1 **(a)**, Model 6 **(b)**, Model 7 **(c)**, Model 12 **(d)**.

Figure 13 presents the T-SNE visualizations of four representative models: Model 1 (baseline without CBAM), Model 6 (single CBAM applied to Conv Block 2-2), Model 7 (optimal configuration with CBAM after Conv Blocks 1-1, 1-2, and 2-1), and Model 12 (CBAM applied to all convolutional layers). The results reveal distinct clustering patterns across the models.

Model 1 shows considerable overlap between the two classes, indicating poor feature separability. Model 6 demonstrates improved clustering, with partial separation between normal and abnormal samples. Model 7 exhibits the most distinct separation, with two clearly defined clusters and minimal intra-class dispersion, aligning with its superior inter-class center distance of 72.0167. In contrast, Model 12 presents an irregular and overlapping distribution, suggesting that excessive CBAM usage may lead to overfitting and less discriminative feature learning.

These observations corroborate the quantitative findings and further confirm that Model 7 not only achieves the highest classification accuracy but also learns the most separable feature space. The use of T-SNE provides compelling visual evidence that supports the effectiveness of selectively integrating CBAM into early and mid-level convolutional layers.

### 4.4 Optimal model analysis

Among the twelve experimental models, Model 7 demonstrated the optimal performance for heart sound classification. First, Model 7 achieved the highest accuracy (98.66%) among all configurations, significantly outperforming both single-CBAM and multi-CBAM models. Second, it exhibited the lowest loss value (0.0344), indicating superior prediction consistency during both training and validation phases. Additionally, in the T-SNE visualization results (Figure 11), Model 7 achieved the largest inter-class center distance (72.0167), suggesting excellent feature separability between normal and abnormal heart sounds.

Further analysis reveals that by strategically integrating CBAM modules after Conv Block 1-1, 1-2, and 2-1, Model 7 effectively enhances mid- and low-level feature representations while avoiding the overfitting issue observed in Model 12, where CBAM was applied to all convolutional layers. The ablation study further validates this configuration: compared to no CBAM (Model 1), CBAM only at deeper layers (Model 9), and CBAM applied to all layers (Model 12), Model 7 consistently achieved superior results across accuracy, loss, and feature separability.

In summary, Model 7 establishes itself as the best-performing heart sound classification model in this study, excelling across accuracy, loss minimization, feature distribution, and architectural optimization.

### 4.5 Ablation study

To further validate the effectiveness of the selected CNN + CBAM configuration, an ablation study was conducted by modifying key architectural components and comparing their classification performance. The following variants were evaluated:

• Variant A (No CBAM): The baseline CNN without any CBAM modules.
• Variant B (CBAM at deeper layers only): CBAM integrated only into the last convolutional stage (Conv Block 3-1 and 3-2).
• Variant C (All layers with CBAM): CBAM inserted into every convolutional block (equivalent to Model 12).
• Variant D (Proposed configuration): CBAM inserted into Conv Block 1-1, 1-2, and 2-1 (Model 7).

As shown in Table 4, the proposed configuration (Variant D) achieved the highest accuracy (98.66%) and the lowest loss (0.0344), outperforming other variants. Variant C, which applied CBAM to all convolutional blocks, suffered from overfitting, leading to reduced performance. These findings demonstrate that selective integration of CBAM into early and mid-level convolutional stages strikes a better balance between feature enhancement and model generalization.

**TABLE 4:** Ablation study results for CBAM Configurations.

Model variant	CBAM placement	Accuracy (%)	Loss
A	Model 1 (None)	92.80	0.3565
B	Model 9 (Only last stage)	98.42	0.0445
C	Model12 (All stages)	91.10	0.1915
D	Model7 (1-1, 1-2, 2-1)	98.66	0.0344

### 4.6 Comparison of different classification algorithms

To contextualize the performance of the proposed CNN + CBAM framework, a comparison with related studies using the same PhysioNet CinC 2016 dataset is presented in Table 5. The proposed CNN + CBAM framework achieves the highest accuracy (0.9866) among the compared methods, outperforming traditional CNN-based approaches. This improvement can be attributed to the following factors:

(1) Adaptive Attention Mechanisms: CBAM’s dual attention mechanisms enable the model to focus on critical spectral features while suppressing irrelevant regions.
(2) Effective Layer Selection: The strategic placement of CBAM in early and mid-level convolutional layers optimizes feature extraction without overfitting.
(3) Minimal Manual Feature Engineering: The proposed method relies on spectrograms as input, reducing the need for manual feature extraction and segmentation.

**TABLE 5:** Comparison of different classification algorithms.

No.	References	Method	Performance of macc
1	Wu et al. (2019)	CNN	0.8981
2	Rubin et al. (2016)	CNN	0.8340
3	Chen et al. (2019)	CNN	0.9481
4	Cheng et al. (2019)	CNN	0.9616
5	Browne and Ghidary (2003)	1D-CNN and 2D-CNN	0.9610/0.9529
6	Katal et al. (2025)	VGG-16/LSTM	0.9644/0.92
7	Our study	CNN + CBAM	0.9866

These results underscore the potential of attention-based architectures for heart sound classification and highlight the importance of optimizing CBAM placement for specific datasets and tasks.

### 4.7 Evaluation results of model 7 on the independent test set

To further evaluate the generalization capability of Model 7 in real-world scenarios, we assessed its performance on an independent test dataset. A total of 12,378 samples were used for training and validation, while the test set was constructed from the PhysioNet 2022 database, ensuring full separation from the training data. Based on a 9:1 ratio between the training (including validation) and test sets, 1,294 heart sound samples were randomly selected to form the test set.

**FIGURE 14:**
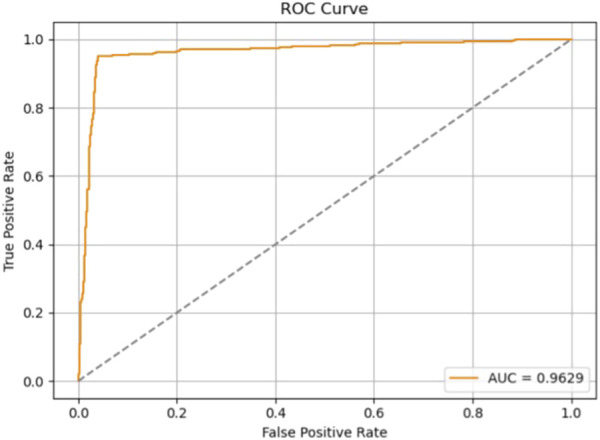
ROC curve of Model 7 on the independent test set.

**FIGURE 15:**
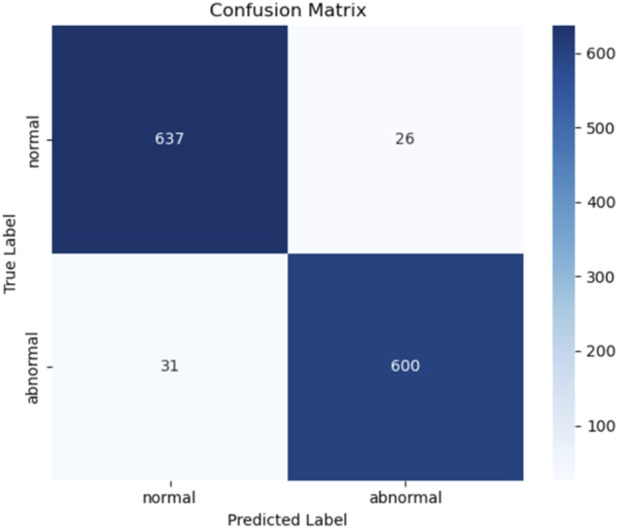
Confusion matrix of Model 7 on the independent test set.

The evaluation results are presented in Table 6 and Figure 14. On the test set, Model 7 achieved an accuracy of 95.6%, sensitivity of 95.09%, precision of 95.85%, F1-score of 95.47%, and an area under the ROC curve (AUC) of 96.29%. Figure 15 shows the confusion matrix, providing a clear visualization of the classification outcomes for normal and abnormal heart sounds. Among the 1,294 test samples, the model accurately identified the majority of both classes, with a low misclassification rate, reflecting strong feature discrimination and classification balance.

**TABLE 6:** Test performance of Model 7 on the independent dataset.

Metric	Value
Accuracy	0.956
Sensitivity (Recall)	0.9509
Precision	0.9585
F1_score	0.9547
AUC	0.9629

In summary, Model 7 maintained excellent performance on the independent test set, with high scores across all evaluation metrics. These findings confirm the model’s robustness and generalization ability, highlighting its potential for application in real-world clinical decision support systems.

In addition, the total inference time for 1,294 heart sound samples was measured to be 0.47 s, corresponding to an average of approximately 0.36 milliseconds per sample. This demonstrates the model’s high efficiency and suitability for real-time clinical applications.

### 4.8 Computational efficiency and practicality

To evaluate the practical deployment potential of our proposed Model 7, we conducted a detailed analysis of its computational efficiency in comparison with two commonly used deep learning baselines—VGG16 and ResNet50. Specifically, we report four key metrics: number of trainable parameters, training time per epoch, average inference time per heart sound sample and frames per second (FPS) (Table 7).

**TABLE 7:** Comparison of computational efficiency across models.

Model name	Number of parameters	Training time/Epoch(s)	Testing time/A heart sample (msec)	Frames per second (FPS)
VGG16	19,440,450	146.13	14.67	76.25
ResNet 50	61,771,778	508.34	13.63	21.92
Model 7(Ours)	588,644	10	0.363	1,114.0

All experiments were conducted on an Apple M4 Mac mini (16 GB RAM, macOS 15.4 Sequoia). The development environment included Python 3.12, TensorFlow 2.17. The datasets used for training and testing times were consistent with those used in this study, ensuring the accuracy and stability of the evaluation results.

Compared to VGG16 and ResNet50, Model 7 demonstrates significant computational efficiency. Specifically, Model 7 reduces the number of parameters by over 97%, leading to a substantial decrease in computational complexity compared to VGG16 and ResNet50. Additionally, Model 7 achieves an inference speed of 1114 FPS, significantly outperforming the other two models, which highlights its promising potential for real-time heart sound analysis tasks.

Despite its compact structure, Model 7 maintains competitive classification accuracy, as shown in Section 4.4. This balance between speed and performance is attributed to the use of a lightweight CNN backbone enhanced with CBAM attention, which selectively emphasizes informative features without incurring high computational costs.

While larger models like ResNet50 may offer marginal gains in feature representation, their significantly higher training and inference costs make them less practical for real-world deployment, particularly in resource-constrained environments such as wearable medical devices or embedded systems. Our results highlight that Model 7 offers the best trade-off between computational complexity and classification accuracy, confirming its suitability for efficient and scalable deployment in intelligent auscultation applications.

## 5 Discussion

This study explores the application of the Convolutional Block Attention Module (CBAM) within a CNN-based framework for heart sound classification, aiming to optimize the number and placement of CBAM modules. Several key findings emerged from the experiments:

First, the selective use of CBAM significantly improved the model’s ability to extract meaningful features from spectrogram representations of heart sounds. The dual attention mechanisms—channel and spatial—enable the model to focus on diagnostically relevant regions while suppressing irrelevant or noisy information.

Second, the ablation experiments confirmed that the effectiveness of CBAM is highly dependent on its placement. While strategic placement improved performance, indiscriminate integration of CBAM into all layers (as in Model 12) resulted in overfitting and reduced generalization. In contrast, Model 7, which applied CBAM after Conv Blocks 1-1, 1-2, and 2-1, achieved the best overall performance, with an accuracy of 98.66% in cross-validation and 95.6% on an independent test set. Furthermore, to mitigate overfitting, an early stopping mechanism was incorporated during model training. This approach halted training when performance on the validation set plateaued, thereby enhancing the model’s generalizability. This supports the hypothesis that attention mechanisms are most effective when integrated into early and mid-level layers.

Third, the T-SNE visualizations clearly illustrated differences in feature space across models. Model 7 achieved the most distinct clustering, with minimal intra-class overlap and a high inter-class center distance, further validating its discriminative power. In contrast, Model 1 and Model 12 showed less effective separation, indicating insufficient feature learning or overfitting.

Compared to other CNN-based approaches, the proposed method achieved superior results, largely due to the synergy between attention-driven feature refinement and well-designed architecture. However, limitations remain. The evaluation primarily relied on the PhysioNet 2016 dataset, and while the test set from PhysioNet 2022 improved robustness, broader clinical validation is still needed. Additionally, although CBAM is lightweight, its use increases computational cost, which may affect deployment in resource-constrained environments.

To evaluate the impact of window length on model performance, we conducted preliminary experiments comparing 5-second and 10-second segmentation windows. The results showed that longer windows introduced additional noise, increased memory consumption, and slightly degraded model performance, with a decrease of approximately 15% in F1 score. Moreover, when using overlapping windows, there is a potential risk of data leakage between the training and validation sets. Based on these findings, we adopted a 5-second non-overlapping window, which achieves a better trade-off between signal completeness, diagnostic sufficiency, and model generalization. While segmenting heart sound recordings into 5-second intervals may raise concerns about missing features beyond this window, we designed the segmentation strategy to balance diagnostic coverage with computational efficiency. Clinical consultations and prior research indicate that pathological features such as murmurs and arrhythmias typically recur across multiple cardiac cycles and are thus likely to be captured within a 5-second interval. To further ensure signal quality, we excluded incomplete tail segments and those shorter than 5 s to avoid introducing boundary artifacts, which could mislead the model during training. Overall, this segmentation approach enhances both model robustness and classification accuracy by ensuring the integrity and diagnostic relevance of the input data.

In addition to its high classification performance, the proposed model shows strong potential for real-world clinical applications. Its fast inference speed (<0.5 s per case) enables near real-time feedback, making it suitable for deployment in digital stethoscopes or mobile diagnostic tools to assist physicians during auscultation, especially in primary care or resource-limited settings. Moreover, the model’s reliance on spectrogram inputs simplifies integration into existing digital health workflows. Future work will focus on validating the model across multiple datasets and clinical scenarios, exploring lightweight attention variants, and developing model compression and acceleration techniques to further support real-time deployment on embedded or portable devices.

## 6 Conclusion

This study successfully demonstrates the potential of integrating the Convolutional Block Attention Module (CBAM) into a convolutional neural network (CNN) for improving heart sound classification accuracy. By leveraging spatial and channel attention mechanisms, the proposed CNN + CBAM framework achieved a classification accuracy of 98.66%, outperforming traditional CNN-based methods on the same dataset. The results highlight the importance of strategic CBAM placement, with early to mid-level convolutional layers proving optimal for enhancing feature extraction without overfitting. However, the study also identifies key limitations, including dataset size constraints and increased computational complexity due to CBAM integration. These challenges underscore the need for further research to enhance the generalizability and practicality of the proposed approach. Future work should focus on expanding the dataset to include diverse heart sound samples, developing lightweight attention mechanisms, and validating the framework in real-world clinical applications. By addressing these limitations, the CNN + CBAM framework has the potential to significantly advance automated heart sound analysis and contribute to improved clinical diagnostics.

## Data Availability

The original contributions presented in the study are included in the article/supplementary material, further inquiries can be directed to the corresponding author.
